# Vegetarianism

**Published:** 1888-04-21

**Authors:** Joseph Knight

**Affiliations:** Secretary of the Vegetarian Society. 75, Princess Street, Manchester.


I am pleased to see in your issue of March 31st so fair an acknowledgment of the usefulness of vegetarian advocacy as tending to aid in the improved dietary of large numbers of people. It is certain that the vegetable kingdom offers a very wide range of food products for selection, and that in combination with such animal products as milk and eggs (these being at individual discretion a part of the vegetarian dietary) pleasant, satisfying, and healthful meals can be obtained without resorting to the use of animal flesh. Should any of your readers desire to know more of the vegetarian system, I shall be pleased to send a few papers to any inquirer on receipt of request.

Joseph Knight,
Secretary of the Vegetarian Society.
75, Princess Street, Manchester.

